# Brain Permeable AMP-Activated Protein Kinase Activator R481 Raises Glycaemia by Autonomic Nervous System Activation and Amplifies the Counterregulatory Response to Hypoglycaemia in Rats

**DOI:** 10.3389/fendo.2021.697445

**Published:** 2021-12-17

**Authors:** Ana M. Cruz, Katie M. Partridge, Yasaman Malekizadeh, Julia M. Vlachaki Walker, Paul G. Weightman Potter, Katherine R. Pye, Simon J. Shaw, Kate L. J. Ellacott, Craig Beall

**Affiliations:** ^1^ Institute of Biomedical and Clinical Sciences, College of Medicine and Health, University of Exeter, Exeter, United Kingdom; ^2^ Rigel Pharmaceuticals Inc., South San Francisco, CA, United States

**Keywords:** AMPK, hypoglycemia, glucagon, hypoglycemic clamp, glucose homeostasis, counterregulation

## Abstract

**Aim:**

We evaluated the efficacy of a novel brain permeable “metformin-like” AMP-activated protein kinase activator, R481, in regulating glucose homeostasis.

**Materials and Methods:**

We used glucose sensing hypothalamic GT1-7 neuronal cells and pancreatic αTC1.9 α-cells to examine the effect of R481 on AMPK pathway activation and cellular metabolism. Glucose tolerance tests and hyperinsulinemic-euglycemic and hypoglycemic clamps were used in Sprague-Dawley rats to assess insulin sensitivity and hypoglycemia counterregulation, respectively.

**Results:**

*In vitro*, we demonstrate that R481 increased AMPK phosphorylation in GT1-7 and αTC1.9 cells. In Sprague-Dawley rats, R481 increased peak glucose levels during a glucose tolerance test, without altering insulin levels or glucose clearance. The effect of R481 to raise peak glucose levels was attenuated by allosteric brain permeable AMPK inhibitor SBI-0206965. This effect was also completely abolished by blockade of the autonomic nervous system using hexamethonium. During hypoglycemic clamp studies, R481 treated animals had a significantly lower glucose infusion rate compared to vehicle treated controls. Peak plasma glucagon levels were significantly higher in R481 treated rats with no change to plasma adrenaline levels. *In vitro*, R481 did not alter glucagon release from αTC1.9 cells, but increased glycolysis. Non brain permeable AMPK activator R419 enhanced AMPK activity *in vitro* in neuronal cells but did not alter glucose excursion *in vivo*.

**Conclusions:**

These data demonstrate that peripheral administration of the brain permeable “metformin-like” AMPK activator R481 increases blood glucose by activation of the autonomic nervous system and amplifies the glucagon response to hypoglycemia in rats. Taken together, our data suggest that R481 amplifies the counterregulatory response to hypoglycemia by a central rather than a direct effect on the pancreatic α-cell. These data provide proof-of-concept that central AMPK could be a target for future drug development for prevention of hypoglycemia in diabetes.

## Introduction

Achieving more time in the target blood glucose (BG) range is a daily challenge for people with diabetes. This can become increasingly challenging with tightening glycemic control using insulin treatment, which increases the risk of hypoglycaemia. Moreover, disease progression and frequent exposure to hypoglycaemia can lead to impaired awareness of and defective counterregulatory responses (CRR) to hypoglycaemia (1).

AMP-activated protein kinase (AMPK) has emerged as a central component of cellular energy sensing over the past two decades. The enzyme is a heterotrimeric complex composed of α, β and γ-subunits, with the α-subunit containing the catalytic domain (2). There are two isoforms of the α-subunit, AMPKα1 and AMPKα2, with the latter isoform having a more prominent role in glucose sensing (3–5). This enzyme plays an important role in regulating whole body energy homeostasis through its actions in the hypothalamus (6) and pancreas (7, 8). Previous studies have shown that direct pharmacological activation of AMPK in the ventromedial nucleus of the hypothalamus (VMH), an important hypoglycemia-sensing brain region (9), increases the response to hypoglycemia in healthy (10), recurrently hypoglycemic and diabetic BB rats (11) by increasing hepatic glucose production (HGP) with or without concomitant increases in glucagon and adrenaline levels. Moreover, suppression of AMPK activity using shRNA diminishes the glucagon and adrenaline response to hypoglycemia (12). Recurrent glucoprivation in rats leads to attenuated AMPK activation in hypothalamic nuclei during hypoglycemia (13), suggesting, at least in part, that recurrent hypoglycemia (RH) may lead to defective CRR through suppression of hypothalamic AMPK activity. Importantly, previous studies have, thus far, only used direct injection of AMPK activators into the brain. Rigel Pharmaceuticals (CA, USA) has developed novel AMPK activating compounds with a similar mechanism of action to metformin [complex I inhibition (14)] but with greater potency. One novel compound, R481, is brain permeable and has a positive brain:plasma distribution. We assessed the effect of R481 on glucose homeostasis and used this novel compound to test the hypothesis that peripheral delivery of a brain-permeable AMPK activator may improve the CRR to hypoglycemia.

## Research Design and Methods

### Reagents

**Figure 1:**
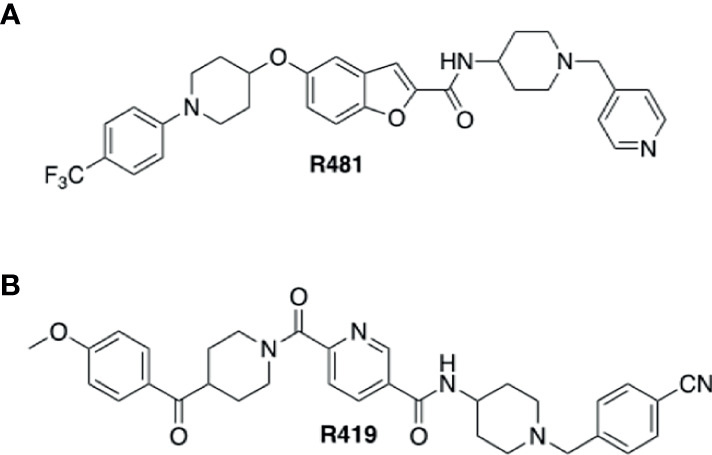
Structures of indirect AMPK activators R481 and R419. Chemical structures of brain permeable R481 **(A)** and non-brain permeable R419 **(B)** indirect AMPK activators.

R481 and R419 were kindly gifted by Rigel Pharmaceuticals Inc (San Francisco, USA). Chemical structures for compounds are shown in **Figure 1**. SBI-0206965 was purchased from Cayman Chemical, hexamethonium bromide from Sigma Aldrich, 50% glucose solution from Centaur Services, and Novo Nordisk Actrapid insulin was purchased from Covetrus.

### Cell Culture

Immortalized GT1-7 mouse hypothalamic cells were a kind gift from Pamela Mellon, Salk Institute, San Diego, California, USA. GT1-7 cells were cultured in growth medium, as previously described (15), and experiments conducted at physiological brain glucose levels (2.5 mmol/L) in experimental medium (**Table 1**). The murine pancreatic α-cell line αTC1 clone 9 (referred to as αTC1.9) was a kind gift from Hannah Welters, University of Exeter, UK. αTC1.9 cells were cultured in growth medium at physiological peripheral glucose levels (5.5 mmol/L) and experiments conducted in experimental medium, KBH buffer or XF medium (**Table 1**). Cell lines were confirmed as mycoplasma free using a commercial kit (MycoAlert, Lonza, Slough, UK).

**Table 1:** Media composition for *in vitro* experiments.

Cell line	Medium	Components
GT1-7	Growth medium	High glucose (25 mmol/L) DMEM (D5671, Sigma) supplemented with 10% FBS (102070-106 Gibco), 4% L-glutamine (ThermoFisher) and 2% penicillin/streptomycin (pen/strep; 100 U/ml; 100 µg/ml; Gibco)
GT1-7	Plating medium	Glucose-free DMEM (11966, ThermoFisher) with 2% L-glutamine supplemented with 7.5 mmol/L glucose, 10% FBS and 2% pen/strep
GT1-7	Experimental medium	Glucose-free DMEM, serum free, supplemented with 2.5 mmol/L glucose
αTC1.9	Growth/plating medium	Glucose free DMEM supplemented with 5.5 mmol/L glucose, 10% FBS (102070-106), 0.02% wt/vol fatty acid free BSA, 0.34 mmol/L mixed essential amino acids, 1 mmol/L pyruvate, 15 mmol/L HEPES and 2% pen/strep
αTC1.9	Experimental medium	Glucose-free DMEM, serum free, supplemented with 1 mmol/L glucose
αTC1.9	Kreb’s-Ringer bicarbonate HEPES (KBH)	In sterile ddH_2_O, 130 mmol/L NaCl, 3.6 mmol/L KCl, 1/5 mmol/L CaCl_2_, 0.5 mmol/L KH_2_PO_4_, 0.5 mmol/L MgSO_4_, 2 mmol/L Na_2_CO_3_, 10 mmol/L HEPES and 0.1% wt/vol fatty acid free BSA, pH 7.4
αTC1.9	Seahorse XF medium	XF DMEM pH 7.4 supplemented with 2.5 mmol/L sodium pyruvate and 2 mmol/L L-glutamine with 0.5 mmol/L glucose or glucose free.

### Immunoblotting

Cells were grown to 60-80% confluence in 60 mm round petri dishes. Following drug treatment, cell lysates were collected for protein quantification using the Bradford method (16). For tissues, brains were dissected from healthy Sprague Dawley rats following administration with R481 (20 mg kg^-1^; i.p), R419 (20 mg kg^-1^; i.p) or vehicle (0.5% HPMC, 0.1% TWEEN-80; i.p) during glucose tolerance tests (2 g kg^-1^) 120 minutes following drug administration and snap frozen in liquid nitrogen. 2 mm sections encompassing the medial basal hypothalamus (MBH) were cut using the rat brain matrix (A1TO 1mm World Precision instruments) and MBH dissected with a scalpel. Protein was extracted from the region of interest by mechanical homogenisation, in lysis buffer, and quantified using the Bradford method. Extracted protein was separated using SDS-PAGE and transferred to nitrocellulose membranes. Immunoreactivity for total and phosphorylated protein was detected and semi-quantified using infrared fluorescence on the Licor Odyssey scanner. Primary antibodies used were: pThr172 AMPK (1:1,000; catalogue #2531), AMPKα (total AMPK 1:1000, catalogue #2535), pSer79 acetyl CoA carboxylase (ACC; 1:1000; catalogue #3661) from Cell Signalling Technologies, total ACC (1:1,000; catalogue #05-1098) from Merck Millipore and β-actin (1:10,000; catalogue #NB600-501) from Biotechne.

### Determination of ATP Concentrations

GT1-7 cells were cultured in 96-well plates overnight and intracellular ATP concentrations were measured using the ATPlite two-step assay (PerkinElmer, UK) as per manufacturer’s instructions and as previously described (17).

### Measurement of Glucagon Release

For glucagon assessments, αTC1.9 cells were seeded in 12-well plates overnight, incubated in serum-free medium for 2 hrs and treated for 1 hr with R481 (50 nmol/L) or vehicle in KBH buffer (**Table 1**) supplemented with 0.5 mmol/L glucose. Media was collected for glucagon quantification using glucagon ELISA (Mercodia, Uppsala, Sweden) and cells lysed for protein quantification. Data analyzed by 4-parameter logistic curve analysis.

### Assessment of Cellular Metabolism

Measurement of basal oxygen consumption rate (OCR), extracellular acidification rate (ECAR) and glycolytic rate assays were performed using the Agilent Seahorse Bioanalyzer according to manufacturer’s instructions with minor modifications (Agilent, United Kingdom). Briefly, αTC1.9 cells were plated on poly-L-lysine (PLL; 4 µg/ml) coated XFe96 microplates (3 x 10^4^ cells/well) in growth medium (**Table 1**) and incubated overnight at 37°C, 5% CO_2_ before experiments. For glucose concentration response experiment, following overnight incubation, cells were washed and incubated in glucose-free XF DMEM at 37°C in non-CO_2_ incubator (de-gas) and treated with R481 (50 nmol/L) or vehicle for 60 minutes. Baseline OCR and ECAR measurements were taken before injection of increasing glucose concentrations (0.1-11.7 mmol/L). For glycolytic rate assays, cells were incubated overnight, as above, and subsequently treated with R481 (50 nmol/L) or vehicle with and without SBI-0206965 (30 µmol/L) for 60 minutes in 0.5 mmol/L glucose containing XF DMEM. Basal ECAR was measured and used to determine glycolytic proton efflux rate (glycoPER), calculated according to manufacturer’s instructions using in house buffer capacity assays. After assays, cells were lysed with NaOH (50 mmol/L) for protein quantification using the method of Bradford. Baseline OCR and ECAR represent an average of the first 4 cycles of each assay.

### Animals

All animals in these studies were male Sprague-Dawley rats (200-350 g) purchased from Charles River Laboratories (Margate, UK). Rats were group-housed in double decker clear plastic cages with ample bedding material and environmental enrichment (wooden chew blocks and a cardboard tube) and maintained on a 12-hour light cycle (06:30 am lights on), temperature 22-23°C, 55% humidity with *ad libitum* access to food (Lab Diet; catalogue number 5LF2) and water. Animals were randomized to treatment groups (computerized randomization) and for most studies, the lead investigator was blinded to treatments (excluding pilot and/or dose finding studies). Rats were fasted for 16 hrs prior to all experiments. All procedures were approved by the University of Exeter Animal Welfare and Ethical Review body and were performed in accordance with the UK Animals Scientific Procedures Act (1986).

### Glucose Tolerance Tests and Feeding Studies

For glucose assessments using SBI-0206965 (3 mg kg^-1^) and hexamethonium (50 mg kg^-1^) or saline vehicle, either drug was delivered 30 minutes before glucose (2 g kg^-1^) ± R481 (5-20 mg kg^-1^); R419 (20 mg kg^-1^) or vehicle (0.5% HPMC + 0.1% TWEEN-80) in a single injection. Substances were administered *via* the intraperitoneal (i.p.) route. Blood glucose was measured at 0, 15, 30, 60 and 120 minutes from a tail vein prick by handheld glucometer (AccuCheck, Roche). Blood samples at 15 minutes were collected from the tail vein using sodium heparin coated capillary tubes. Blood was centrifuged at 5000 rpm for 10 minutes at 4°C and plasma collected and snap frozen in liquid nitrogen for insulin quantification. For feeding studies, food hopper containing chow was weighed after 1-24 hours, depending on the study.

### Hyperinsulinemic Clamp Studies

Male Sprague-Dawley rats (200-300 g) with pre-implanted jugular vein and carotid artery catheters were purchased from Charles River (Margate, UK). Catheters were exteriorized using a dual-channel vascular access button (Instech, USA) and covered using a lightweight aluminium cap, enabling social housing following surgery. This improved body weight curves post-surgery. Catheter patency was maintained by flushing the catheters every 3-5 days with heparinized glucose catheter lock solution. R481 (20 mg kg^-1^; i.p.) or vehicle (0.5% HPMC, 0.1% TWEEN-80; i.p.) were administered following overnight fast, 1 hr prior to the hyperinsulinemic-euglycemic or hypoglycemic clamp. Blood glucose was measured every 5-10 min and larger blood samples for hormone analysis were collected every 30 min from the carotid artery catheter. During euglycemic clamps, animals received a fixed continuous insulin infusion of 20 mU kg^-1^ min^-1^ and a variable dextrose (20% w/v; i.v.) infusion rate to maintain glycaemia at approximately 5.5 mmol/L. To induce hypoglycemia, rats received a bolus insulin infusion of 80 mU kg^-1^ min^-1^ for 10 minutes, followed by a maintenance dose of 20 mU kg^-1^ min^-1^ for the remainder of the clamp. A variable 20% (w/v) dextrose infusion was used to maintain blood glucose levels at approximately 2.8-3 mmol/L at nadir. Six animals were excluded from the study for poor catheter patency issues. Overall patency rates were approximately 85% for animals maintained up to 21 days post-surgery.

### Hormone and Metabolite Analysis

Plasma glucagon, C-peptide and insulin were measured using ELISA (Mercodia, Uppsala, Sweden). Plasma adrenaline was measured using the Demeditec Adrenaline ELISA (Kiel, Germany).

### Statistical Analysis

A one-sample t-test was used to determine significant changes in phosphorylated or total protein expression relative to control in immunoblotting experiments. Unpaired t-tests were used to compare groups in non-normalized immunoblotting data. Blood glucose levels, glucose infusion rates and plasma analytes were analyzed using a two-way ANOVA with repeated measures or mixed-effects analysis in cases where datasets were missing data points. Peak hormone levels were analyzed using an unpaired t-test. Analyses were performed using the GraphPad Prism (Prism 8, GraphPad, La Jolla, CA, USA). Results are expressed as mean ± SEM, with *p < 0.05* considered statistically significant.

## Results

### R481 Activates AMPK in Hypothalamic Neuronal Cells

**Figure 2:**
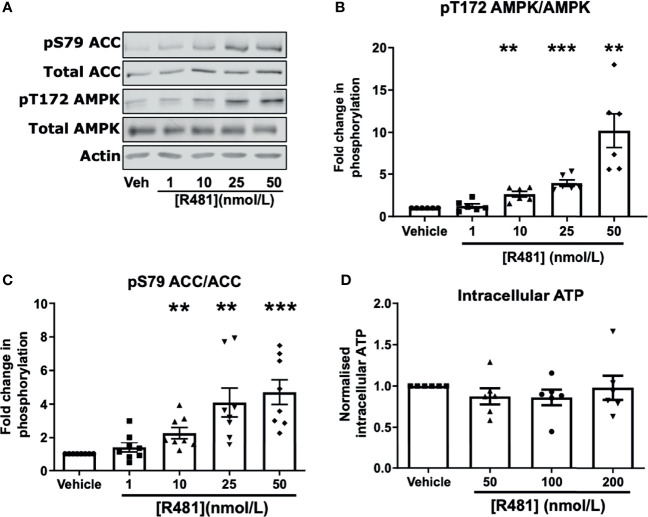
AMPK is activated in GT1-7 hypothalamic neuronal cells by R481. Mouse GT1-7 hypothalamic neurons treated with increasing concentrations of R481 for 30 minutes. **(A)** Representative Western blots for AMPK (pT172), total AMPK, ACC (pS79), total ACC and Actin. Densitometric analysis of the mean pooled data for phospho-AMPK normalized to total AMPK shown in **(B)** (n=6) and phospho-ACC normalized to total ACC in **(C)** (n=8; **P<0.01; ***P<0.001; One-sample t-test in comparison to control). **(D)** Intracellular ATP levels of GT1-7 cells treated with R481, normalized to vehicle (30 minutes; n=6).

To confirm that R481 activated AMPK in neuronal cells, we utilized the mouse hypothalamic glucose-sensing GT1-7 line (3). In GT1-7 cells, treatment for 30 minutes with increasing concentrations of R481 (0-50 nmol/L) increased AMPK phosphorylation at threonine 172 (**Figures 2A, B**). Phosphorylation of the downstream AMPK substrate, ACC, was also significantly increased by R481 (**Figures 2A, C**). Despite AMPK activation, total intracellular ATP levels were not compromised by R481, even at concentrations up to 200 nmol/L (**Figure 2D**). Treatment with R419 (50 nmol/L) did not alter AMPK phosphorylation in GT1-7 cells (**Supplementary Figure 2**) but did increase phosphorylation of ACC, suggesting modest AMPK activation by an AMP-dependent mechanism, as expected for a mild mitochondrial complex I inhibitor.

### R481 Raises Peak Glycaemia Which Is Attenuated by AMPK Inhibitor SBI-0206965 and Abolished by Autonomic Blockade

**Figure 3:**
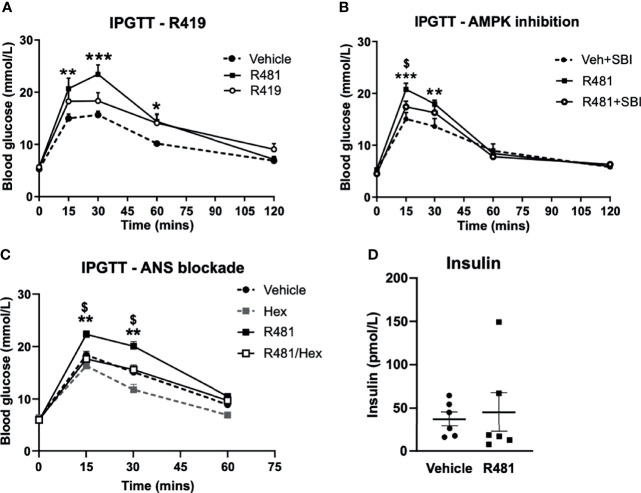
R481 increases peak glucose excursion in a manner that is attenuated by AMPK inhibitor SBI-0206965 and ANS blocker hexamethonium and does not alter insulin levels. Glucose tolerance tests in male Sprague-Dawley rats fasted for 16 hrs. **(A)** Rats were administered glucose (2 g kg-1; i.p.) together with either vehicle (HPMC/Tween-80; n=6), R419 (20 mg kg-1; n=6) or R481 (20 mg kg-1;n=6) Two-way ANOVA with repeated measures, *P(drug)<0.05, ***P(time)<0.001, **P(interaction)<0.01, with Bonferroni’s analysis *P<0.05, **P<0.01, ***P<0.001 for R481 against vehicle; no significant difference between R419 and vehicle groups. **(B)** Rats were administered SBI-0206965 (3 mg kg-1; i.p.) or vehicle 30 minutes before R481 (5 mg kg-1; i.p.) or vehicle (HPMC/Tween-80; i.p.) together with glucose (2 g kg-1; i.p.)(SBI-0206965 n=6; R481 n=10; R481+SBI-0206965 n=8); Two-way ANOVA with repeated measures *P(drug)<0.05, ***P(time)<0.001, ***P(interaction)<0.001 and Bonferroni’s multiple comparisons analysis, **P<0.01, ***P<0.001 for R481 against vehicle, $P<0.05 for R481 *versus* R481+SBI-0206965. **(C)** Rats were treated with hexamethonium (Hex; 50 mg kg-1) or vehicle (saline) 30 minutes before combined administration of glucose (2 g kg-1; i.p.) and R481 (20 mg kg-1; i.p.) or vehicle (HPMC/Tween-80; i.p.); Two-way ANOVA with repeated measures ***P(drug)<0.001, ***P(time)<0.001, ***P(interaction)<0.001 Veh (n=8); Hex (n=3); R481 (n=7); R481/Hex (n=8) with Bonferroni’s analysis **P<0.01 for R481 against vehicle and ^$^P<0.05 for R481 against R481/Hex. **(D)** Plasma insulin levels measured from 15 minute sample of vehicle (n=7) and R481 (n=7) groups in C by ELISA.

Dosing studies in mice demonstrated that R481 rapidly enters the brain (**Supplementary Figure 1A**), displaying a brain:plasma ratio of >3 (**Supplementary Figure 1B**) and increases whole brain AMPK phosphorylation following bolus intravenous infusion in mice (**Supplementary Figure 1C**). In contrast, a related compound R419 did not display significant brain permeability (data courtesy of Rigel Pharmaceuticals. Inc). To determine whether R481 altered glucose tolerance, rats were given a combined intraperitoneal injection of R481 (5-20 mg kg^-1^) and glucose (2 g kg^-1^). R481 treated rats showed significantly higher peak glucose excursions yet glucose levels were not significantly different between groups at 2 hrs post-injection, suggesting effective clearance of glucose. This effect was not reproduced following treatment with non-brain permeable R419 (peak glucose 15.7 ± 1.7 mmol/L in Veh, 23.4 ± 1.8 mmol/L in R481 and 18.3 ± 1.6 in R419 groups; **Figure 3A**). The R481-mediated increase in peak glucose levels was attenuated by pre-treatment with AMPK/Uncoordinated (Unc)-51-like kinase (ULK-1) inhibitor SBI-0206965 (3 mg kg^-1^; peak glucose 20.7 ± 1.2 mmol/L in R481 vs 17.4 ± 1.1 mmol/L in R481+SBI group; **Figure 3B**) (18), which has demonstrated brain penetrance and been shown to inhibit autophagy in the brain (19). To examine whether the autonomic nervous system (ANS) played a role in raising glucose levels, we pre-treated rats with pan autonomic blocker hexamethonium (50 mg kg^-1^) prior to glucose tolerance testing. Glucose excursion in hexamethonium treated rats was not altered by R481 treatment, suggesting that autonomic blockage completely abolished the effect of R481 on glycaemia (peak glucose 22.3 ± 0.7 mmol/L R481 vs 17.6 ± 1.1 mmol/L in R481 + Hex group; **Figure 3C**). At peak glucose levels (15 minutes), insulin concentrations were comparable between vehicle and R481 treated rats (37.2 ± 8.3 pmol/L Veh vs 45.3 ± 22.6 pmol/L R481; **Figure 3D**). Together with R419 data this supports a central action of R481 in regulating glycaemia. On examination of AMPK phosphorylation in the medial basal hypothalamus of R481 and R419 treated rats, there was modestly but not significantly increased levels compared to vehicle controls (>10% increase), with no change seen following R419 treatment (**Supplementary Figure 3**).

As hypothalamic AMPK activation increases feeding (6) and leptin-induced repression of feeding requires inhibition of AMPK (20), we postulated that a brain permeable AMPK activator may increase feeding behavior. However, R481 treatment did not alter *ad libitum*, fasting or hypoglycemia-induced feeding in rats (**Supplementary Figure 4**).

### R481 Does Not Alter Glucose Infusion Rates During a Hyperinsulinemic-Euglycemic Clamp

**Figure 4:**
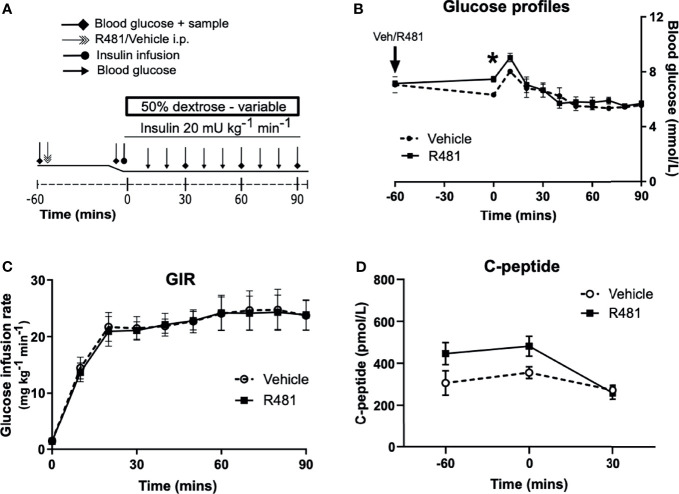
R481 does not alter glucose infusion rate during hyperinsulinemic-euglycemic clamp or alter endogenous insulin secretion. Hyperinsulinemic-euglycemic clamps performed in male Sprague-Dawley rats. **(A)** Study design. **(B)** Blood glucose profiles (Vehicle n=8, R481 n=8; 20 mg kg^-1^; i.p.). No overall drug effect P(drug)>0.05; mixed-effects analysis of repeated measures, but *P<0.05 for R481 against vehicle at t=0 minutes, using Bonferroni’s *post-hoc* test. **(C)** Glucose infusion rate (GIR; mg kg^-1^ min^-1^) during the clamp using a 50% dextrose solution (Vehicle n=8; R481 n=8). **(D)** Plasma C-peptide measured by ELISA (Vehicle n=8; R481 n=7).

R481 (20 mg kg^-1^) or vehicle were administered 60 minutes before insulin infusion (see study design, **Figure 4A**; blood glucose target: 5.5 mmol/L). Baseline glucose levels were moderately increased in R481 treated animals (t = -60 minutes; 7.2 ± 0.2 mmol/L vs t = 0; 7.5 ± 0.2 mmol/L), compared to vehicle group, as blood glucose levels decreased in the latter following drug treatment (t= -60 minutes 7.1 ± 0.6 to t= 0 6.3 ± 0.2 mmol/L). This produced a significant relative increase in blood glucose at the start of the clamp in R481 treated animals (n=8; **Figure 4B**). Glucose levels were well-matched during the last 30 minutes of the clamp (**Figure 4B**), with no difference in the glucose infusion rate (GIR; **Figure 4C**). The levels of C-peptide were not different between groups (**Figure 4D**).

### R481 Reduces the GIR and Increases Glucagon Levels During A Hyperinsulinemic-Hypoglycemic Clamp

**Figure 5:**
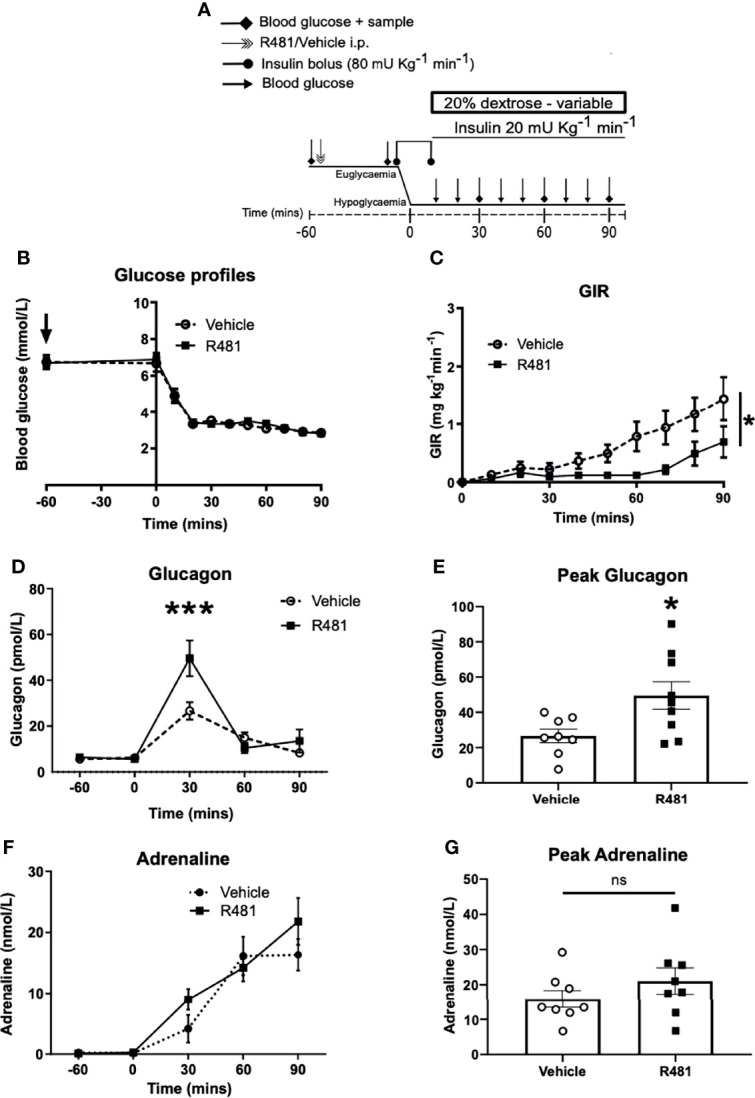
R481 delays exogenous glucose requirements during hyperinsulinaemic-hypoglycaemic clamp by augmenting glucagon levels during hypoglycaemia. Hyperinsulinaemic-hypoglycaemic clamps performed in male Sprague-Dawley rats. **(A)** Study design. Animals were fasted for 16 hrs. R481 (20 mg kg^-1^; i.p.) or vehicle (HPMC/Tween-80; i.p.) were administered 60 minutes before the start of the clamp. **(B)** Blood glucose profiles before and during clamp (Vehicle n=10; R481 n=8). **(C)** Glucose infusion rates (GIR; mg kg^-1^ min^-1^) during the clamp using a 20% dextrose solution. *P(drug)<0.05, ***P(time)<0.05, *P(interaction)<0.05; Two-way ANOVA with repeated measures. **(D)** Plasma glucagon profile with peak shown in **(E)**, measured by ELISA (Vehicle n=9; R491 n=9; *P<0.05, unpaired t-test). **(F)** Plasma adrenaline profile with peak shown in **(G)**, measured by ELISA (Vehicle n=8; R481 n=8; ns, not significant).

To determine the potential influence of central AMPK activation, using R481, on CRR, we induced hypoglycemia (2.8 mmol/L) during a 90 minute clamp study (Study design **Figure 5A**). Glucose levels during the clamp were well-matched between vehicle and R481-treated rats (**Figure 5B**). Exogenous glucose infusion required to maintain hypoglycemia was significantly lower in R481-treated animals (**Figure 5C**). Peak plasma glucagon levels were significantly higher in the R481 treated group (**Figures 5D, E**). Adrenaline levels were not different between groups (**Figures 5F, G**).

### R481 Activates AMPK and Enhances Glucose Utilization in Pancreatic α-Cells During Low Glucose Exposure

**Figure 6:**
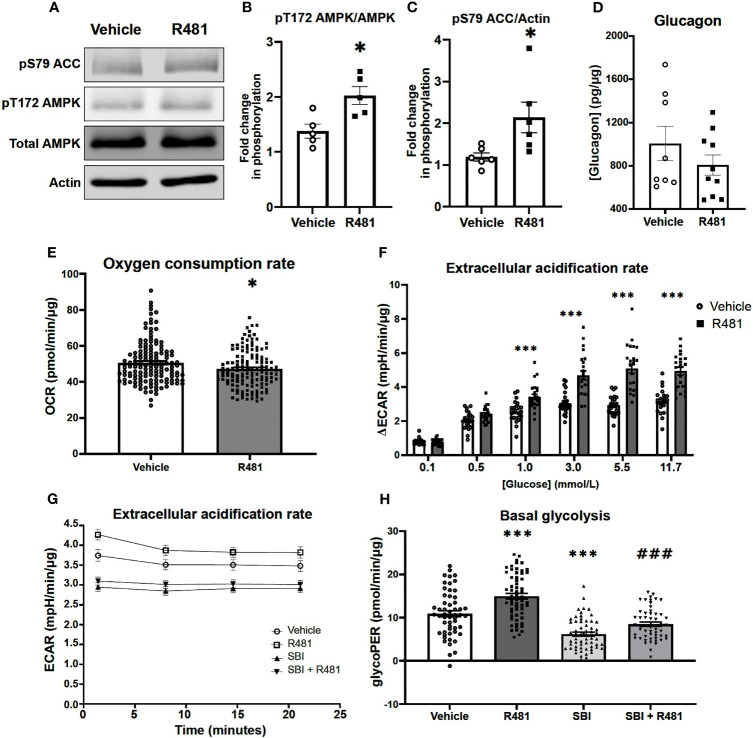
R481 activates AMPK and enhances glycolysis during low glucose in murine pancreatic α-cells. Murine αTC1.9 pancreatic α-cells treated with R481 during hypoglycemia. **(A)** Representative Western blot for AMPK (pT172), total AMPK, ACC (pS79), total ACC and Actin. Densitometric analysis of the mean pooled data for phospho-AMPK normalized to total AMPK in **(B)** (n=5) and phopho-ACC normalized to total ACC in **(C)** (n=6) for cells treated with R481 (50 nmol/L) or vehicle in 1 mmol/L glucose (*P<0.05 unpaired t-test). **(D)** Glucagon measured by ELISA in αTC1.9 cells treated with R481 (50 nmol/L) or vehicle for 1 hr in 0.5 mmol/L glucose (Veh n=8; R481 n=10). Measurement of baseline oxygen consumption rate in **(E)** (n= 138; *P<0.05; unpaired t-test) prior to and change in extracellular acidification rate in **(F)** (n= 24; delta reflects change from baseline glucose-free levels) following acute treatment with glucose (0.1-11.7 mmol/L) in cells treated for 1 hr with R481 (50 nmol/L) or vehicle. **(G)** Representative trace of basal ECAR for cells treated with R481 (50 nmol/L) or vehicle with or without SBI-0206967 (Vehicle n=54; R481 n=60, SBI n=59, R481+SBI n=54) in 0.5 mmol/L glucose with assessment of glycolytic proton efflux rate (glycoPER) in **(H)** (***P<0.001 compared to vehicle; ^###^P<0.001 compared to R481 group).

Given that R481 significantly augmented peak glucagon levels, murine αTC1.9 cells were used as a model of pancreatic α-cell to examine the effect of R481 on AMPK activation, glucagon secretion and cellular metabolism during low glucose exposure. Treatment of αTC1.9 cells for 60 minutes with R481 (50 nmol/L) at 1 mmol/L glucose significantly enhanced AMPK phosphorylation at threonine 172 (**Figures 6A, B**) and ACC phosphorylation at serine 79 (**Figures 6A, C**). Glucagon release following 60 minute treatment with R481 (50 nmol/L) or vehicle at 0.5 mmol/L glucose was not different between groups (Veh 1044 ± 141.3; R481 832.6 ± 68.0 pg/mg; **Figure 6D**). To assess changes to cellular metabolism in these glucose sensing cells, αTC1.9 cells were treated for 60 minutes with R481 (50 nmol/L) in the absence of glucose; oxygen consumption rate (OCR) was measured, and cells were treated with increasing glucose concentrations to assess changes to extracellular acidification rate (ECAR) as a proxy for glycolysis. Baseline OCR levels were modestly but significantly decreased in R481 treated cells compared to vehicle, as expected (**Figure 6E**). The glucose-dependent increase in ECAR was augmented by R481, which tended to increase at 0.5 mmol/L and was significantly elevated in R481 treated cells from 1 mmol/L to 11.7 mmol/L glucose (**Figure 6F**). Finally, to test the effect of AMPK inhibition on the R481-mediated ECAR response, cells were treated with R481 (50 nmol/L) with or without AMPK inhibitor SBI-0206965 (30 µmol/L) for 60 minutes in 0.5 mmol/L glucose. The R481-mediated increase in basal ECAR was blunted by treatment with SBI-0206965 (**Figure 6G**). To distinguish whether this represents mitochondria derived proton acidification or glycolysis derived acidification, glycoPER, or glycolytic proton efflux rate was measured. Cells treated with R481 showed significantly higher glycolytic rates compared to vehicle and this increase was completely abolished by SBI-0206965 treatment. Moreover, SBI-0206965 treatment alone significantly lowered basal glycoPER, most likely driven by endogenous increases in AMPK activity during low glucose exposure (**Figure 6H**).

## Discussion

AMPK activators have been developed for glucose lowering in Type 2 Diabetes (T2D), largely by acting on skeletal muscle to promote glucose disposal (21, 22). The R481 analogue, R419 (non-brain permeable), activates AMPK in skeletal muscle and increases insulin sensitivity in high-fat fed mice (23). We demonstrate here that R481 (brain permeable) raises glucose levels during GTTs, without negatively impacting glucose clearance, in a manner that was attenuated by AMPK inhibition and completely abolished by autonomic blockade, suggesting R481 acts centrally to mediate these effects. Previous studies have shown that genetic or pharmacological suppression of hypothalamic AMPK activity decreases hepatic glucose production [HGP (24)]. Conversely, direct infusion of non-specific AMPK activator AICAR into the hypothalamus stimulates HGP (24, 25). In addition, fructose ingestion has been shown to increase hypothalamic AMPK activity and increase HGP (26). In combination, these studies indicate that hypothalamic AMPK activity regulates HGP and supports the suggestion that R481 may require hypothalamic AMPK activation to stimulate HGP, possibly *via* the brain stem circuit, as demonstrated previously (25). Our data concur with these earlier observations and extend them by demonstrating that peripheral delivery of a brain permeable AMPK activator can increase glycaemia. Defining the specific brain nuclei and the neural circuitry involved will be important in future studies.

Kume and colleagues (27) demonstrated that activation of hypothalamic AMPK suppressed first phase glucose-stimulated insulin secretion (GSIS) through autonomic innervation of α-adrenergic pancreatic nerves. This was suggested to be a physiological response to promote glucose delivery to the brain during fasting (27), a mechanism that may also occur during hypoglycemia. However, in our study, R481 did not alter glucose-stimulated insulin secretion nor did it alter C-peptide levels during the clamp studies, suggesting that R481 does not suppress basal insulin secretion and likely increases glycaemia by stimulating HGP, as has been previously reported following viral and pharmacological manipulation of hypothalamic AMPK activity (24–26).

We postulated that R481 treatment may increase the GIR during the euglycemic clamp by enhancing skeletal muscle glucose uptake. However, the GIR during the euglycemic clamp was not altered by R481. This suggests that the compound is not having a direct metformin-like effect in the liver to suppress HGP. However, given that R481 has a positive brain:plasma ratio, it is plausible that there is insufficient compound accumulation in the liver to significantly change glucose production directly. Moreover, we examined glucose homeostasis following a single injection of the drug in lean rats and saw no direct evidence of glucose lowering, again suggesting that there is little effect in peripheral tissues such as skeletal muscle or adipose tissue. Whether acute and/or chronic R481 treatment would have a glucose lowering effect in rats fed a high-fat diet (HFD) remains to be determined. However, a previous study demonstrated that the R481 analogue R419, given chronically to HFD fed mice (23), enhanced skeletal muscle glucose uptake and insulin sensitivity. It is important to highlight that our data suggest the transient increase in blood glucose levels by R481 was not mediated by a change to insulin secretion or sensitivity. Moreover, despite the fact that glucose levels peaked higher in the R481 treated animals during the GTT, glucose levels at 2 hours post glucose administration were not different from controls, suggesting that glucose clearance was not altered and possibly greater compared to vehicle treated animals. Using glucose tracers to determine the rates of glucose appearance and disappearance will be important going forward to closely examine HGP and skeletal muscle glucose uptake.

In previous studies with non-diabetic rats, direct pharmacological activation of AMPK in the VMH using AICAR amplified HGP during hypoglycemia, without altering glucagon or adrenaline release (10). In line with this, basal glucagon and adrenaline release in our study were not altered by R481. Importantly, previous studies have demonstrated that pharmacological and genetic activation of AMPK in pancreatic α-cells was sufficient to stimulate glucagon release (7, 8). Taken together with our data, it is plausible that R481 may have direct actions in pancreatic α-cells to augment glucagon release. To assess this further we treated pancreatic α-cells *in vitro* with R481 during exposure to low glucose. Our data suggest that treatment with R481 increases AMPK activation at low glucose and, like other indirect AMPK activators, appears to cause mild mitohormesis, evidenced by decreased basal oxygen consumption. However, R481 did not alter glucagon secretion during low glucose treatment, at least in this cell model. In contrast, R481 treatment significantly enhanced glucose utilization by amplifying glucose-dependent glycolytic rate, which would be expected to result in suppression of glucagon release. This R481-mediated increase in glycolysis was completely abolished by pre-treatment with allosteric AMPK inhibitor SBI-0206965, indicating the R481-induced changes in cellular metabolism are most likely AMPK mediated, although it should be noted that SBI-0206965 can also inhibit ULK1 at these concentrations (18). Taken together, these actions of R481 suggest that the compound does not alter glucagon secretion through a pancreatic α-cell-mediated mechanism and provides evidence that *in vivo*, R481 may raise glucagon levels during hypoglycemia by a centrally-mediated pathway.

In streptozotocin-induced diabetic rats, VMH AICAR injection can augment both glucagon and adrenaline responses during hypoglycemia (11). Given that both hyperglycemia and recurrent hypoglycemia/glucoprivation suppress hypothalamic AMPK activation (6, 13) and that direct genetic suppression of VMH AMPK expression/activity suppresses the glucagon and adrenaline responses to hypoglycemia (12), it is plausible that hypothalamic AMPK activity is blunted in diabetes, leading, at least in part, to defective CRR. In our study, R481 may activate an AMPK-ANS-HGP axis, whilst also increasing plasma glucagon levels to better defend against hypoglycemia. Delineating the central *versus* peripheral actions of pharmacological AMPK activation during hypoglycemia requires further study. It will also be interesting to determine the effect of R481 when given chronically and by a method that provides slower release of R481, such as with an osmotic mini-pump, as the action of R481 was limited to 2-2.5 hours when delivered by the intraperitoneal route. One of the limitations of our study was that we were unable to conclusively demonstrate increased AMPK phosphorylation or activity in brains from rats treated with R481. We have demonstrated a trend towards increased AMPK phosphorylation following R481 but not R419 treatment in the medial basal hypothalamus. However, we did not observe statistically significant differences, which may be due to increased basal AMPK activation as a consequence of hypoxia during anaesthesia prior to tissue extraction (28).

Importantly, our data highlight that the likely net effect of brain AMPK activation is to increase glucose delivery to the brain (27), indicating that, at the level of the whole organism, central AMPK activation may supersede peripheral activation in a hierarchical manner, akin to that suggested for subcellular pools of AMPK (29). This raises the interesting possibility that centrally biased AMPK activating drugs could be used to raise blood glucose levels and peripheral activators to reduce glycaemia, meaning that a drug or combination of drugs that activate central and peripheral AMPK could be used to attenuate the peaks and troughs in blood glucose seen in diabetes. Our data also raise the interesting possibility that if metformin were to activate AMPK in the brain, this possible glucose-raising action could compete with the effect of metformin in the liver to suppress gluconeogenesis (30). The blood brain barrier is more leaky in T2D/obesity (31–33) and several clinical studies have shown possible central effects of metformin (34, 35), suggesting that metformin may enter the brain at efficacious levels in some circumstances. Taken together, this could raise the possibility that metformin “failure” in T2D could be caused by metformin-induced activation of brain AMPK, stimulating ANS-mediated HGP, competing against the actions of the drug in the liver.

In summary, our data indicate that peripheral delivery of a brain permeable AMPK activator raises glycaemia, likely to protect brain function. We provide proof-of-concept that pharmacological activation of central AMPK may be a suitable therapeutic target for amplifying the defense against hypoglycemia. This requires testing in rodent models of T1D and T2D and in rodents with defective CRR where careful optimization of the dose to amplify CRR without worsening fasting/fed hyperglycemia will be needed. To be clinically useful, any anti-hypoglycemic drug would need to be taken prior to the unpredictable development of hypoglycemia. A drug with an optimized pharmacodynamic/pharmacokinetic profile permitting dosing, for example, before bedtime, could be taken to prevent the development of nocturnal hypoglycemia. It will also be interesting to determine whether central AMPK activating drugs could be used as a treatment for severe hypoglycemia to promote rapid recovery of blood glucose levels. In conclusion, development of brain permeable allosteric activators of AMPK could be useful for the prevention/treatment of hypoglycemia in diabetes.

## Data Availability Statement

The raw data supporting the conclusions of this article will be made available by the authors, without undue reservation.

## Ethics Statement

The animal study was reviewed and approved by University of Exeter Animal Welfare and Ethical Review Board.

## Author Contributions

A.M.C, K.M.P, Y.M., J.M.V.W., P.G.W.P, K.R.P, and C.B. researched data. A.M.C., K.M.P, J.M.V.W., S.J.S., K.L.J.E., and C.B., contributed to study design. All authors contributed to writing the manuscript and approved the final version. C.B. conceived the study, had access to all the data collected at the University of Exeter and takes responsibility for the accuracy and integrity of the data.

## Funding

This study was funded by: a JDRF Innovative grant (1-INO-2016-214-A-N) to CB and KE; a JDRF postdoctoral fellowship (3-PDF-2020-941-A-N) to PW, a Diabetes UK RD Lawrence Fellowship to CB (13/0004647); a Diabetes UK PhD studentship to CB and KE for KMP. (18/0005914); a Society for Endocrinology early career grant to CB and a British Society for Neuroendocrinology practical skills grant to CB. AC was funded by a University of Exeter Medical School PhD studentship.

## Author Disclaimer

The views expressed are those of the author(s) and not necessarily those of the NHS, the NIHR or the Department of Health and Social Care.

## Conflict of Interest

SJS is an employee and shareholder of Rigel Pharmaceuticals, Inc.

The remaining authors declare that the research was conducted in the absence of any commercial or financial relationships that could be construed as a potential conflict of interest.

## Publisher’s Note

All claims expressed in this article are solely those of the authors and do not necessarily represent those of their affiliated organizations, or those of the publisher, the editors and the reviewers. Any product that may be evaluated in this article, or claim that may be made by its manufacturer, is not guaranteed or endorsed by the publisher.
