# Supplementary figures and images for: Brain Permeable AMP-Activated Protein Kinase Activator R481 Raises Glycaemia by Autonomic Nervous System Activation and Amplifies the Counterregulatory Response to Hypoglycaemia in Rats
Source: Front Endocrinol (Lausanne). 2021 Dec 17;12:697445. doi: 10.3389/fendo.2021.697445 (PMC8718766; doi:10.3389/fendo.2021.697445)

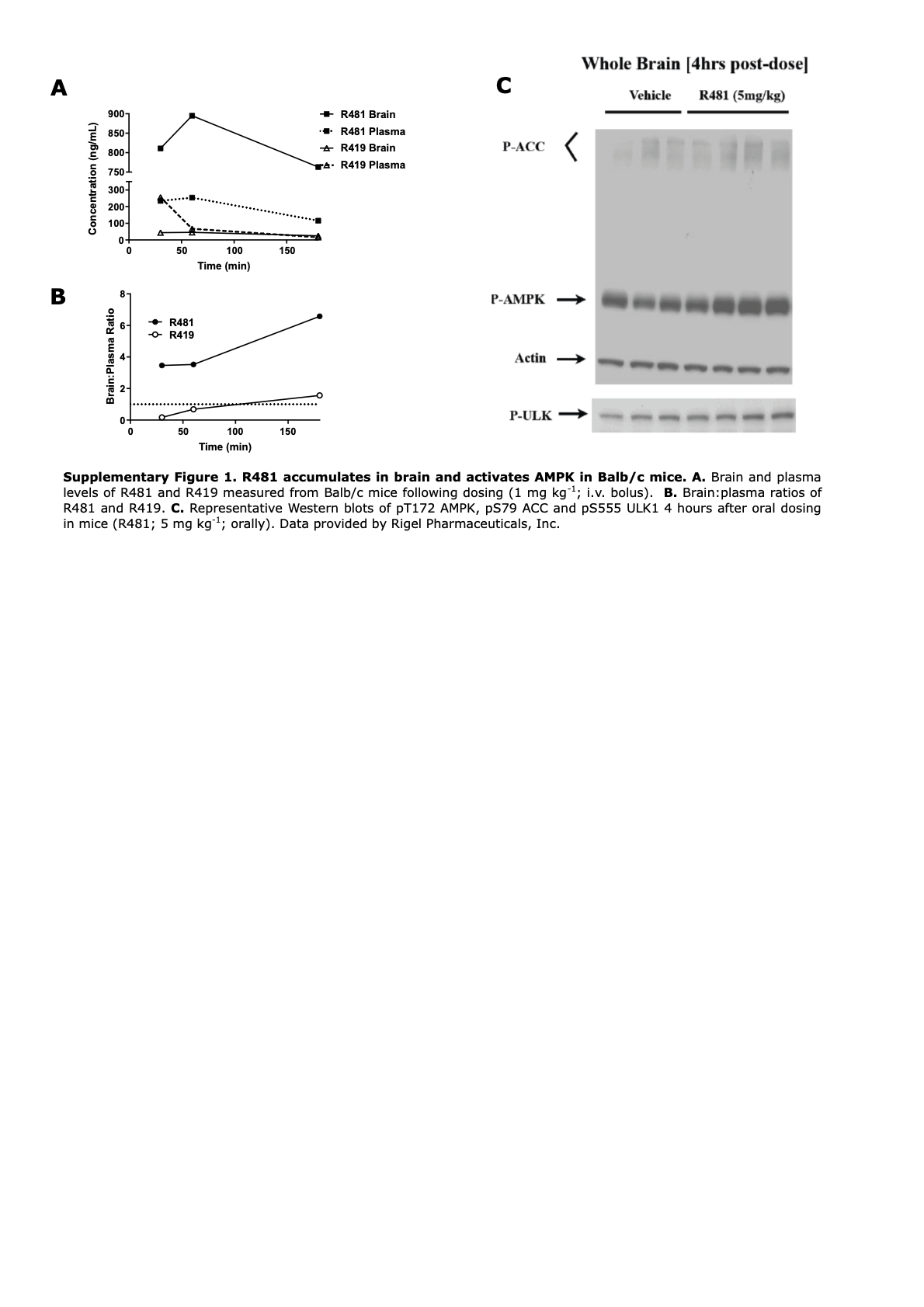

Supplement: Supplementary file 1 [file Image_1.tiff]

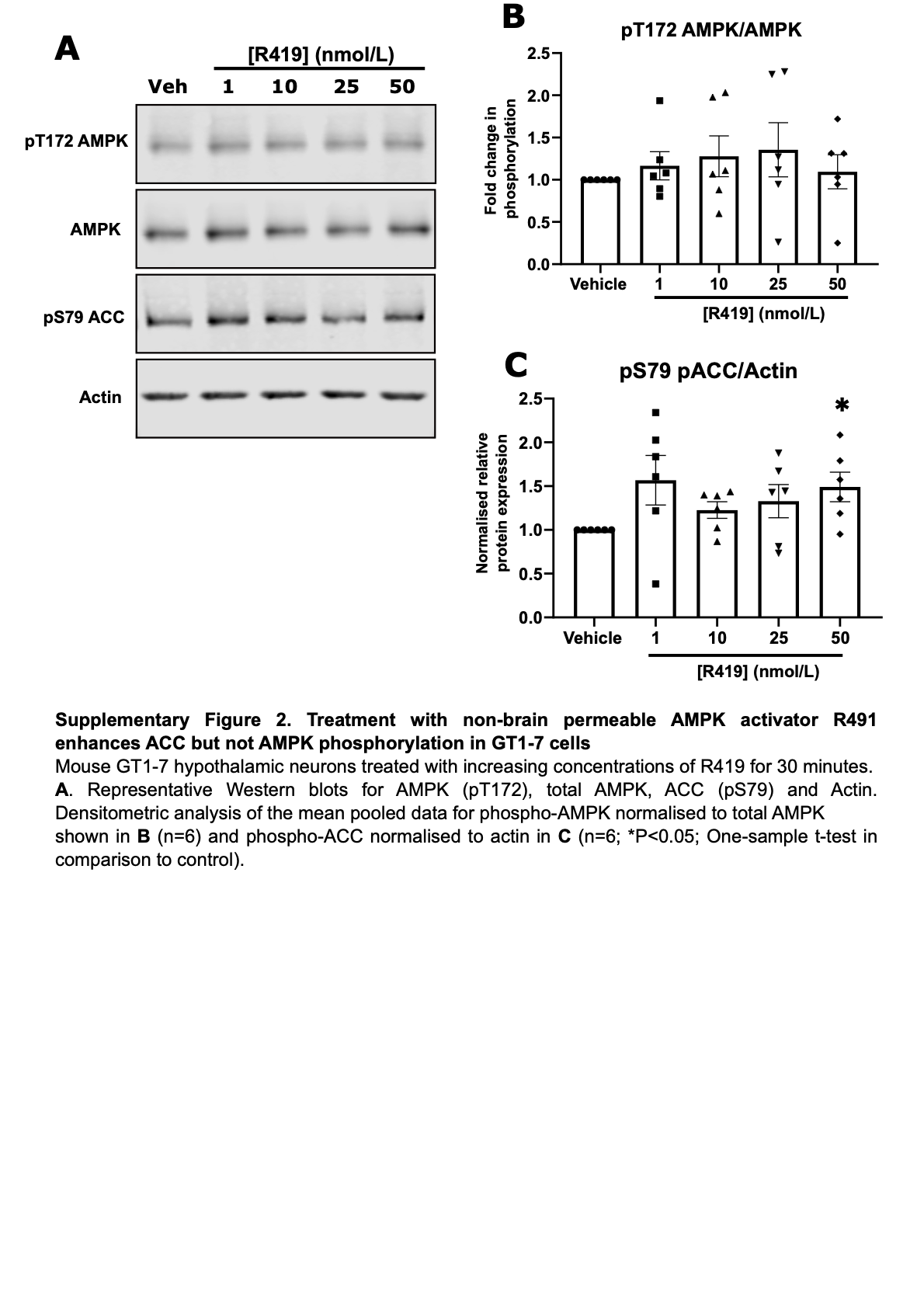

Supplement: Supplementary file 2 [file Image_2.tiff]

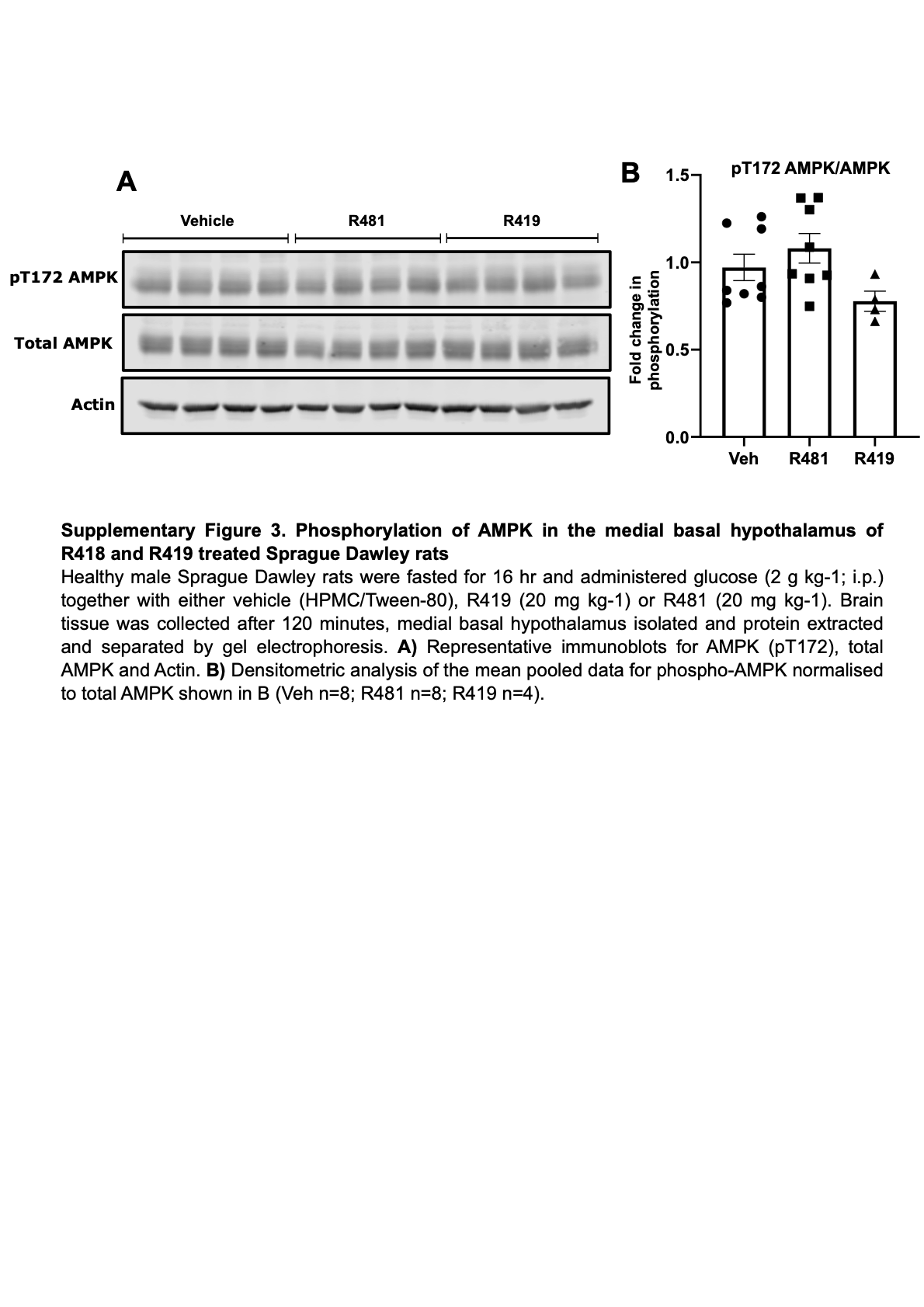

Supplement: Supplementary file 3 [file Image_3.tiff]

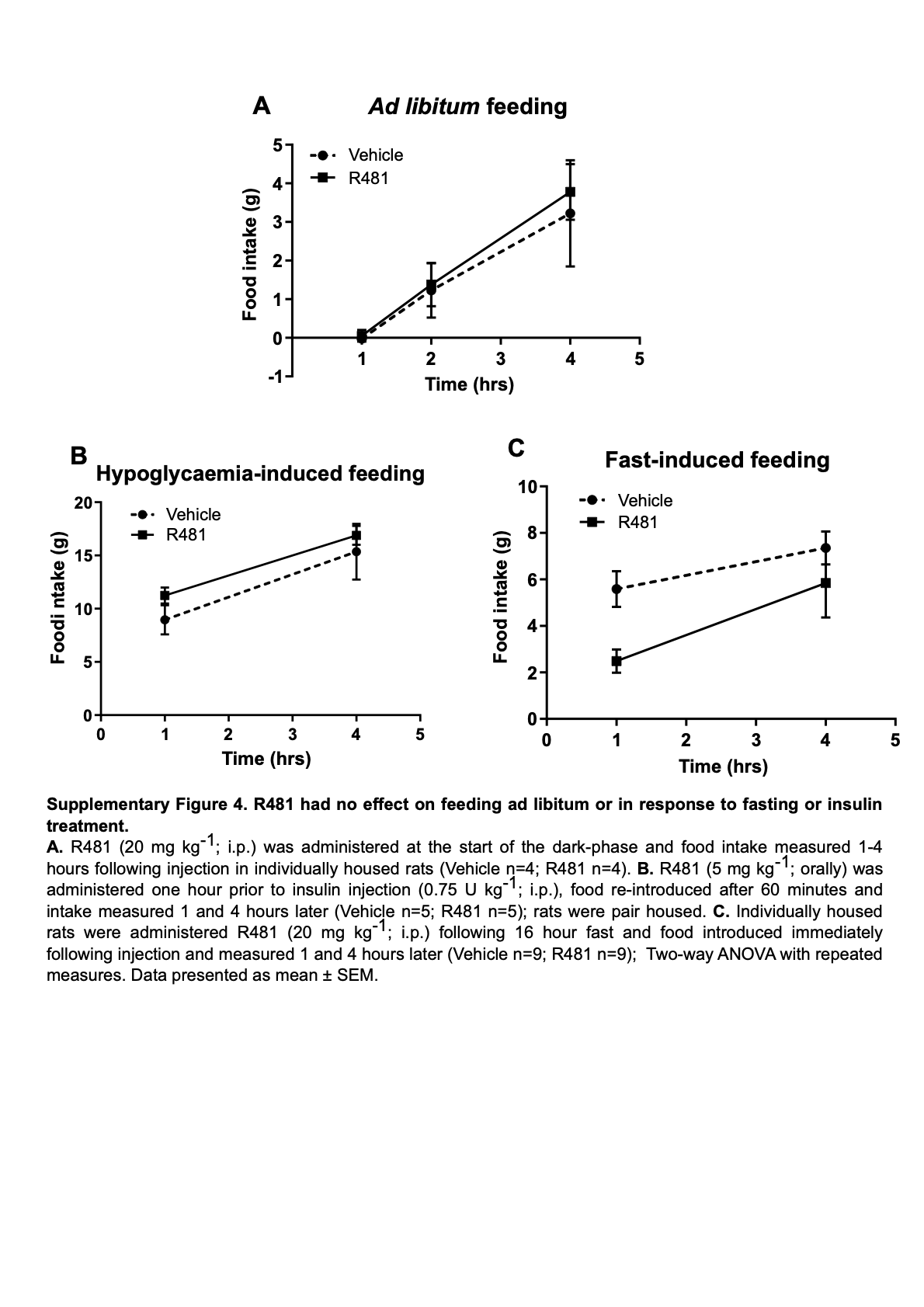

Supplement: Supplementary file 4 [file Image_4.tiff]
